# Transcriptome Sequencing and Development of Novel Genic SSR Markers From *Pistacia vera* L.

**DOI:** 10.3389/fgene.2020.01021

**Published:** 2020-09-09

**Authors:** Harun Karcι, Aibibula Paizila, Hayat Topçu, Ertuğrul Ilikçioğlu, Salih Kafkas

**Affiliations:** ^1^Department of Horticulture, Faculty of Agriculture, Çukurova University, Adana, Turkey; ^2^Pistachio Research Institute, Gaziantep, Turkey

**Keywords:** transcriptome, RNA-seq, eSSR, *Pistacia*, phylogenetic

## Abstract

In this study, we aimed to develop novel genic simple sequence repeat (eSSR) markers and to study phylogenetic relationship among *Pistacia* species. Transcriptome sequencing was performed in different tissues of Siirt and Atl cultivars of pistachio (*Pistacia vera*). A total of 37.5-Gb data were used in the assembly. The number of total contigs and unigenes was calculated as 98,831, and the length of N50 was 1,333 bp after assembly. A total of 14,308 dinucleotide, trinucleotide, tetranucleotide, pentanucleotide, and hexanucleotide SSR motifs (4–17) were detected, and the most abundant SSR repeat types were trinucleotide (29.54%), dinucleotide (24.06%), hexanucleotide (20.67%), pentanucleotide (18.88%), and tetranucleotide (6.85%), respectively. Overall 250 primer pairs were designed randomly and tested in eight *Pistacia* species for amplification. Of them, 233 were generated polymerase chain reaction products in at least one of the *Pistacia* species. A total of 55 primer pairs that had amplifications in all tested *Pistacia* species were used to characterize 11 *P. vera* cultivars and 78 wild *Pistacia* genotypes belonging to nine *Pistacia* species (*P. khinjuk*, *P. eurycarpa*, *P. atlantica*, *P. mutica*, *P. integerrima*, *P. chinensis*, *P. terebinthus*, *P. palaestina*, and *P. lentiscus*). A total of 434 alleles were generated from 55 polymorphic eSSR loci with an average of 7.89 alleles per locus. The mean number of effective allele was 3.40 per locus. Polymorphism information content was 0.61, whereas observed (Ho) and expected heterozygosity (He) values were 0.39 and 0.65, respectively. UPGMA (unweighted pair-group method with arithmetic averages) and STRUCTURE analysis divided 89 *Pistacia* genotypes into seven populations. The closest species to *P. vera* was *P. khinjuk*. *P. eurycarpa* was closer *P. atlantica* than *P. khinjuk*. *P. atlantica*–*P. mutica* and *P. terebinthus*–*P. palaestina* pairs of species were not clearly separated from each other, and they were suggested as the same species. The present study demonstrated that eSSR markers can be used in the characterization and phylogenetic analysis of *Pistacia* species and cultivars, as well as genetic linkage mapping and QTL (quantitative trait locus) analysis.

## Introduction

*Pistacia* L. genus is a member of the Anacardiaceae family that also contains important species such as mango, pepper tree, and sumac (Kafkas, 2006a). The sex habit of *Pistacia* is dioecious with several exceptions (Kafkas et al., 2000). The genus of *Pistacia* consists of 13 or more species (Gundesli et al., 2019), and *Pistacia vera* is believed to be the most ancestral species, whereas the other species probably derived from Zohary (1952) and Kafkas (2019). Currently, the main pistachio producers in the world are Iran, the United States, Turkey, and Syria (FAO, 2019).

The first taxonomic study in the genus *Pistacia* was done morphologically by Zohary (1952). After discovery of different molecular markers, they have been used in *Pistacia* as well. The first detailed molecular study in *Pistacia* was performed based on chloroplast DNA profiles by Parfitt and Badenes (1997). Microsatellites or simple sequence repeats (SSRs) and repeats of 1- to 6-nucleotide-long DNA motifs have high reproducibility, multiallelic character, and extensive tandem repeats in the whole genome (Powell et al., 1996). SSRs have advantages over other marker systems because of their codominant inheritance, suitability for automation, and well-distribution throughout eukaryotic genomes. Recently, SSRs have been widely used in genetic map construction, DNA fingerprinting, genetic diversity, quantitative trait locus (QTL) mapping, and marker-assisted selection (MAS) (Dong et al., 2018; Yang et al., 2018; Zhang et al., 2019).

Genic SSRs or eSSRs are obtained by expression sequence tags that are created by gene transcripts that have been converted into cDNA (Adams et al., 1991). Recently, eSSR markers have been used for identifying plant species because of its design from coding regions (Varshney et al., 2005; Ellis and Burke, 2007). The concern here is that because eSSRs are located within the genes, and more conserved, they may be used for identification of alleles related to some agronomically significant traits (Chen et al., 2017; Dong et al., 2018; Yang et al., 2018). Conventionally, SSR development needs to be labor-intensive, such as cloning DNA and constructing library, and generates particularly less SSRs compared with next-generation sequencing (NGS) technology (Zalapa et al., 2012; Zhang et al., 2012). The advantage of NGS technologies, especially next-generation transcriptome sequencing, provides a large amount of sequences with cost-effective and high-quality data in a short period (Wang et al., 2010; Taheri et al., 2019; Zhang et al., 2019).

RNA sequencing (RNA-seq) is considered to provide information about functional genes, used to detect reliable and high-throughput eSSR markers (Wang et al., 2018; Taheri et al., 2019). Using RNA-seq, eSSRs have been reported in several plant species such as bean (Chen et al., 2015), grape (Huang et al., 2011), coffee (Ferrão et al., 2015), tomato (Zhou et al., 2015), barley (Zhang et al., 2014), cotton (Tabbasam et al., 2014), wheat (Gupta et al., 2003; Gadaleta et al., 2009), cucumber (Hu et al., 2010), walnut (Zhu et al., 2009), and citrus (Chen et al., 2006).

The aims of the study were (i) to develop novel genic SSR markers from transcriptome sequences of pistachio (*P. vera*) and (ii) to determine phylogenetic relationship among *Pistacia* species using novel eSSRs.

## Materials and Methods

### Plants Materials

In this study, RNA isolation and transcriptome sequencing were performed in bud, flower, leaf, shoot, whole nut, pericarp, and kernel of Siirt (female) and Atl (male) cultivars (Supplementary Table 1). The sampled tissues were immediately frozen in liquid nitrogen and stored at -80°C until RNA isolation.

**FIGURE 1:**
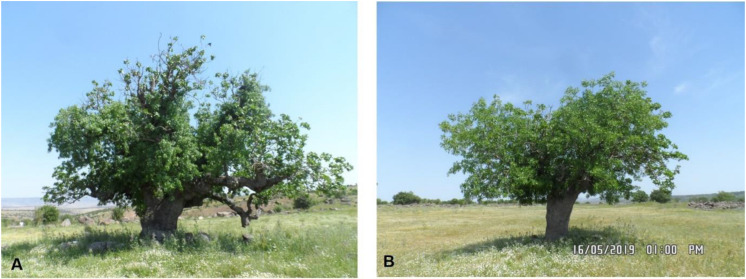
Pictures of two old *P. eurycarpa* genotypes. **(A)** PE-1, **(B)** PE-2.

The genomic DNAs belonging to 11 *P. vera* L., 5 *P. khinjuk* stocks, 13 *P. atlantica* Desf., 5 *P. mutica* F.&M., 3 *P. atlantica* × *P. integerrima* hybrids (UCB1), 8 *P. integerrima* Stewart, 8 *P. chinensis* Bunge, 11 *P. terebinthus* L., 5 *P. palaestina* Boiss., 12 *P. lentiscus* L., and 6 unknown *Pistacia* genotypes were used to verify eSSR markers. The leaf samples from these genotypes were collected from germplasm collections in Çukurova University in Adana, Pistachio Research Institute in Gaziantep. Wild *P. atlantica* and *P. eurycarpa* genotypes growing in the nature in Manisa and Gaziantep provinces were used as well. Two *P. eurycarpa* genotypes were about 500–600 years old in Göbek village (Figure 1 and Supplementary Table 2).

### RNA Extraction, Sequencing, Transcriptome Assembly

Total RNA was extracted from different tissues of pistachio and was sequenced by BGI (Beijing Genomic Institute) using an Illumina (Hi-Seq 2500) NGS platform. The raw reads were first cleaned from adaptors and filtered for low-quality sequences with higher than 20% *Q* value < 20 bases. Those called as “clean reads” were assembled using Trinity software (v2.0.6) (Grabherr et al., 2011). After *de novo* assembly, Trinity sequences were named as transcripts obtained from contigs that were not extended on each ends The transcripts were clustered and obtained final unigenes with TGIGL (Pertea et al., 2003) software. Simple sequence repeats were searched using MIcroSAtelite (MISA) (Haas et al., 2013) search module in all unigenes. The search parameters were set for the detection of dinucleotide, trinucleotide, tetranucleotide, pentanucleotide, and hexanucleotide SSR motifs with a minimum of six, five, four, four, and four repeats, respectively. Primers pairs were designed using online software Batchprimer 3.0 with the standard parameters (You et al., 2008) (Supplementary Table 3). The sequence data have been deposited in the National Center for Biotechnology Information under BioProject accession number PRJNA648340.

### DNA Extraction, Polymerase Chain Reaction Amplification, eSSR Validation

The genomic DNAs of 89 *Pistacia* samples were extracted from fresh leaves using the CTAB method of Doyle and Doyle (1987) with minor modifications (Kafkas et al., 2006). DNA concentrations were measured using a Qubit Fluorimeter (Invitrogen) or were estimated by comparing the band intensity with λ DNA of known concentrations following 0.8% agarose gel electrophoresis and ethidium bromide staining. DNA samples were subsequently diluted to a concentration of 10 ng/μL for eSSR–polymerase chain reaction (PCR).

Initially, a total of 250 randomly selected primer pairs were screened in eight *Pistacia* individuals, and 55 primer pairs were selected for the characterization of 89 *Pistacia* genotypes belonging to 10 *Pistacia* species.

eSSR-PCR was carried out using a three-primer strategy according to the method utilized by Schuelke (2000) with minor modifications (Topçu et al., 2016). PCR was performed in a total volume of 12.5 μL containing 20 ng DNA; 75 mM Tris–HCl (pH 8.8); 20 mM (NH_4_)_2_SO_4_; 2.0 mM MgCl_2_; 0.01% Tween 20; 200 μM each dNTP; 10 nM M13 tailed forward primer at the 5′ end; 200 nM reverse primer; 200 nM universal M13 tail primer (5 TGTAAAACGACGGCCAGT-3) labeled with one of FAM, VIC, NED, or PET dyes; and 0.6 U hot-start Taq DNA polymerase. Amplification was performed in two steps as follows: initial denaturation at 94°C for 3 min, followed by 10 cycles at 94°C for 30 s, 58°C for 45 s, and 72°C for 60 s. The second step included 30 cycles at 94°C for 30 s, 58°C for 45 s, and 72°C for 60 s and a final extension at 72°C for 10 min. After the PCR was completed, the reactions were subjected to denaturation for capillary electrophoresis in an ABI 3130xl genetic analyzer [Applied Biosystems Inc., Foster City, CA, United States (ABI)] using a 36-cm capillary array with POP7 as the matrix (ABI). Samples were fully denatured by mixing 0.5 μL of the amplified product with 0.2 μL of the size standard and 9.8 μL formamide. The fragments were resolved using ABI data collection software 3.0, and SSR fragment analysis was performed with GeneScan Analysis Software 4.0 (ABI).

### Genetic Diversity

The 55 polymorphic eSSR loci were used for the genetic diversity of 11 *P. vera* and 78 wild *Pistacia* genotypes. The number of alleles (Na), the number of effective alleles (Ne), observed (Ho), and expected (He) heterozygosity were calculated using GenAlEx version 6.5 according to Peakall and Smouse (2012). The polymorphism information contents (PICs) of each marker were calculated using PowerMarker software version 3.25 (Liu and Muse, 2005). The SSR bands scored as present (1) or absent (0) consisted of a dendrogram using NTSYSpc v2.21c (Rohlf, 2009) software by unweighted pair-group method with arithmetic averages (UPGMA). A principal coordinate analysis (PCoA) was performed using NTSYSpc v2.21c (Rohlf, 2009).

STRUCTURE 2.3.4 software (Pritchard et al., 2000) was also used to determine the possible number of populations and for the construction of the population structure. Structure analysis computes the proportion of the genome of an individual originating from each interfered population. Possible *K*’s (where *K* is an assumed fixed number of subpopulations in the entire population) were from 1 to 10 with five replications to ensure consistency of results. Ln P(D)s mean possible estimated *K*’s. There are Ln P(D) values for each *K* value. By using Ln P(D) values for every *K* calculate Δ*K* that shows a possible number of populations. Each replication run was conducted with a burn-in period of 100,000 and 100,000 Markov chain Monte Carlo.

## Results

### Sequencing and Assembly

Sixteen transcriptome libraries were constructed from different tissues of Siirt (female) and Atl (male) cultivars, and a total of 374,726,850 clean reads were obtained. A total of 98,831 unigenes were generated by the Trinity software, and the N50 of unigenes was computed as 1,333 bp (Supplementary Table 4). All unigenes were classified according to size of the sequences; 55,101 (55.75%) were 100–500 bp, 18,462 (18.68%) were 500 bp–1 kb, 10,615 (10.74%) were 1–1.5 kb, 6,804 (6.88%) were 1.5–2 kb, and the rest of sequences 7,849 (7.94%) were >2 kb (Table 1).

**TABLE 1:** Length distribution of assembled unigenes.

Nucleotides length	Unigenes
100–500 bp	55,101
500–1 kb	18,462
1–1.5 kb	10,615
1.5–2 kb	6,804
>2 kb	7,849
N50 bp	1,333
Mean length (bp)	776
Max length (bp)	660
Min length (bp)	379
All unigenes	98,831

### Identification and Distribution of SSR Motifs

The MISA search module was used to search for SSRs with the 98,831 unigenes. In total, 37,793 potential genic SSRs were identified from 98,831 unigene sequences, of which 23,485 were mononucleotide repeats (Supplementary Table 5).

**FIGURE 2:**
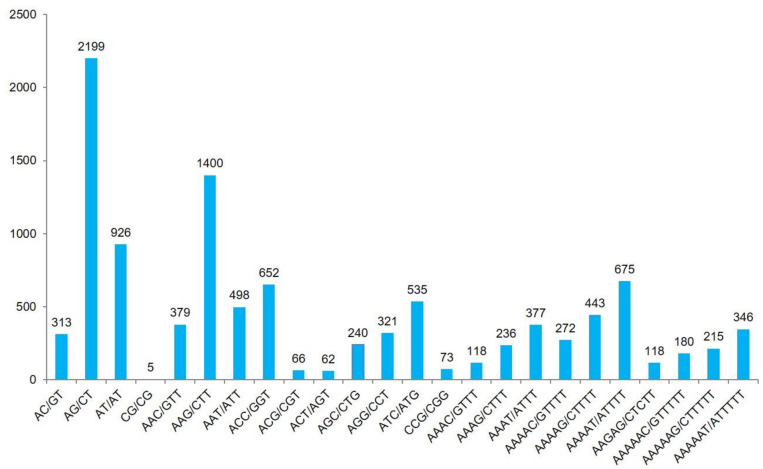
The most abundant types of repeats belonging to dinucleotide, trinucleotide, tetranucleotide, pentanucleotide, and hexanucleotide SSR motifs and the numbers of SSR motifs.

A total of 14,308 dinucleotide, trinucleotide, tetranucleotide, pentanucleotide, and hexanucleotide SSR motifs were detected, and the most abundant type of the repeats was trinucleotide motifs (29.54%), followed by dinucleotide (24.06%), hexanucleotide (20.67%), pentanucleotide (18.88%), and tetranucleotide motifs (6.85%) (Table 2 and Supplementary Table 6). The most abundant repeats were AG/CT (15.4%), AAG/CTT (9.8%), and AT/AT (6.5%). The most abundant tetranucleotide, pentanucleotide, and hexanucleotide repeat motif types were AAAT/ATTT (2.6%), AAAAT/ATTTT (4.7%), and AAAAAT/ATTTTT (2.4%), respectively (Figure 2).

**TABLE 2:** Number of repeats; number of dinucleotide, trinucleotide, tetranucleotide, pentanucleotide, and hexanucleotide SSR motifs; total number of SSR motifs; percentage of SSR motifs; and total number of SSR motifs in *P. vera*.

No. of repeats	Dinucleotide	Trinucleotide	Tetranucleotide	Pentanucleotide	Hexanucleotide
4	0	0	0	2,286	2,467
5	0	0	736	361	486
6	0	2,354	201	54	4
7	1,228	1,139	43	1	0
8	714	590	0	0	0
9	449	136	0	0	0
10	414	3	0	0	0
11	345	3	0	0	0
12	238	1	0	0	0
13	51	0	0	0	0
14	1	0	0	0	0
15	2	0	0	0	0
16	0	0	0	0	0
17	1	0	0	0	0
Total	3,443	4,226	980	2,702	2,957
%	24.06	29.54	6.85	18.88	20.67
Total of repeats	14,308				

### Validation of SSRs

The 250 genic SSR primer pairs were designed from assembled short RNA sequences. The 250 eSSR primer pairs were screened in eight *Pistacia* species (*P. vera*, *P. khinjuk*, *P. atlantica*, *P. mutica*, *P. integerrima*, *P. chinensis*, *P. terebinthus*, and *P. lentiscus*) by agarose gel electrophoresis (1.5%). In total, 233 were amplified at least in one of the *Pistacia* species, and 82 were amplified in all tested eight *Pistacia* species (Supplementary Table 7). Of them, 55 eSSR primer pairs were used for characterization of 89 *Pistacia* accessions because of their polymorphism and having amplification in the tested *Pistacia* species.

### Functional Annotation and Classification

A total of 98,831 assembled unigenes were aligned to different universal databases. Annotation demonstrated that 52,839 unigenes (53.46%) were found important in the Nr database, 49,727 (50.31%) in the Nt database, and 34,419 (34.82%) in the Swiss-Prot database. The annotation of 58,401 (59.09%) unigenes was successfully achieved in at least one of the six public databases (Supplementary Table 8).

**FIGURE 3:**
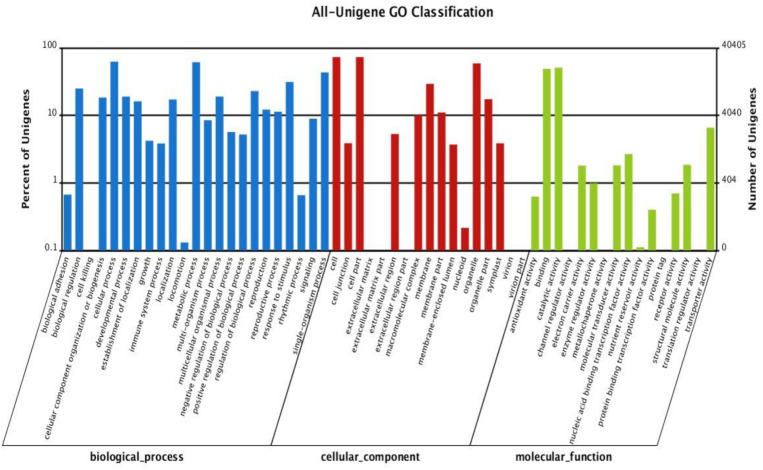
Gene ontology classification of assembled unigenes. The 40,405 matched unigenes were classified into three functional categories: biological process, cellular component, and molecular function.

According to Gene Ontology (GO) analysis, there were 40,405 annotated unigenes classified into three functional categories: biological process, cellular component, and molecular function. The largest classes in biological process were “cellular process” (25,241), “metabolic process” (24,781), and “single organism process” (17,507), respectively. The cellular component category mostly consists of proteins involved in “cell” (29,529), “cell part” (29,528), and “organelle” (23,825). The highest classes in the molecular function category were detected as “binding” (19,780), “catalytic activity” (20,554), and “transporter” (2,654), respectively (Supplementary Table 9 and Figure 3).

**FIGURE 4:**
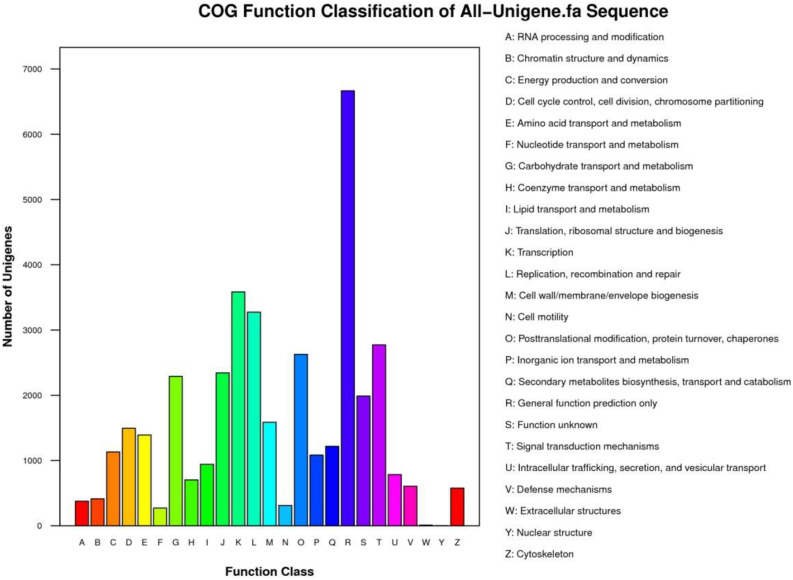
COG classification of all unigenes according to 25 specific categories.

The unigenes were aligned to COG database, and classification of 19,349 (19.58%) unigenes was divided into 25 specific categories (Supplementary Table 10 and Figure 4). Among these categories, “general function prediction only” (6,665), “transcription” (3,584), “replication, recombination and repair” (3,276), “signal transduction mechanisms” (2,774), “posttranslational modification, protein turnover, chaperones” (2,627), and “translation, ribosomal structure, and biogenesis” (2,343) formed the largest groups.

**FIGURE 5:**
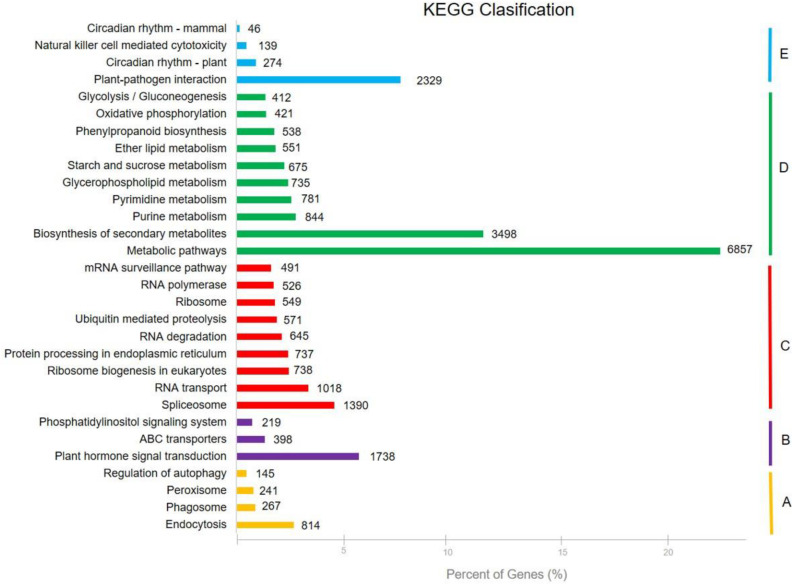
Five KEGG biochemical pathways that obtained from 128 KEGG pathways: **(A)** Cellular Processes, **(B)** Environmental Information Processing, **(C)** Genetic Information Processing, **(D)** Metabolism, and **(E)** Organismal Systems.

KEGG Orthology (KO) database was used for metabolic pathway analysis. In the results constructed in a total 128 KEGG pathways, 30,746 unigenes were categorized into five KEGG biochemical pathways: Cellular Processes (A), Environmental Information Processing (B), Genetic Information Processing (C), Metabolism (D), and Organismal Systems (E). The pathways involving the highest number of unique transcripts were “metabolic pathways” (6,857), followed by “biosynthesis of secondary metabolites” (3,498), “plant-pathogen interaction” (2,329), and “plant hormone signal transduction” (1,738) (Supplementary Table 11 and Figure 5).

### Genetic Diversity in *Pistacia*

Diversity analyses were performed in 11 pistachio cultivars (*P. vera*) and in 78 wild *Pistacia* accessions. Allele ranges, number of alleles (Na), effective number of alleles (Ne), PICs, and expected and observed heterozygosities of 55 eSSR loci are given in Table 3.

**TABLE 3:** Mean of population genetic parameters SSR loci in each of *Pistacia* species.

Population	No. of alleles	Polymorphic allele (%)	Polymorphic/monomorphic markers	Na	Ne	Ho	He	PIC
*Pistacia*	434	100.00%	55/1	7.89	3.40	0.39	0.65	0.61
*P. vera*	150	92.73%	51/4	2.73	1.93	0.38	0.40	0.34
*P. khinjuk*	152	94.55%	52/3	2.76	2.17	0.61	0.49	0.42
*P. eurycarpa*	105	61.82%	34/21	1.91	1.76	0.48	0.33	0.27
*P. atlantica*	191	94.55%	52/3	3.47	2.12	0.34	0.40	0.36
*P. mutica*	117	65.45%	36/19	2.13	1.71	0.30	0.30	0.25
UCB-1	109	70.91%	39/16	1.98	1.83	0.56	0.36	0.30
*P. integerrima*	104	69.09%	38/17	1.89	1.72	0.63	0.34	0.27
*P. chinensis*	151	90.91%	50/5	2.75	1.82	0.34	0.35	0.31
*P. terebinthus*	180	83.64%	46/9	3.27	2.02	0.34	0.38	0.33
*P. palaestina*	114	61.81%	34/21	2.07	1.63	0.28	0.28	0.31
*P. lentiscus*	131	69.09%	38/17	2.38	1.65	0.24	0.29	0.27
Unknown	163	96.36%	53/2	2.96	2.10	0.45	0.46	0.41

A total of 434 alleles were amplified by 55 eSSR loci, ranging from 4 to 20 per locus. The highest number of allele (Na = 20) was amplified by the CUPVEST3927 locus. The effective number of allele ranged from 1.36 (CUPVEST9032) to 12.31 (CUPVEST3927). The highest observed heterozygosity (Ho = 0.92) was obtained from the CUPVEST1146 locus. The average expected heterozygosity was calculated as 0.65, ranging from 0.26 (CUPVEST9032) to 0.92 (CUPVEST3927). The PICs ranged from 0.25 (CUPVEST9032) to 0.92 (CUPVEST3927), with an average of 0.61 (Table 4).

**TABLE 4:** Novel genic SSR genetic diversity values in 89 *Pistacia* individuals: allele ranges, number of allele (Na), number of effective allele (Ne), observed heterozygosity (Ho), expected heterozygosity (He), and (PIC) values of 55 loci.

SSR loci	Repeat motifs	Allele ranges (bp)	Na	Ne	Ho	He	PIC
CUPVEST1146	(AAAGA)_4_	150–173	9	3.37	0.92	0.7	0.65
CUPVEST1313	(GGTGGT)_4_	167–216	12	7.04	0.56	0.86	0.84
CUPVEST1566	(GTT)_8_	168–190	7	4.80	0.26	0.79	0.76
CUPVEST1855	(TTTAT)_4_	124–156	9	5.01	0.59	0.80	0.77
CUPVEST1887	(CAT)_8_	154–179	10	4.68	0.49	0.79	0.75
CUPVEST1981	(ATGGGC)_4_	105–145	10	3.26	0.50	0.69	0.66
CUPVEST2110	(TCTTC)_4_	162–205	7	2.48	0.21	0.6	0.53
CUPVEST2125	(ATC)_8_	151–170	15	5.32	0.57	0.81	0.79
CUPVEST2611	(TGA)_7_	105–122	6	1.42	0.06	0.30	0.28
CUPVEST2680	(CCTTC)_4_	93–146	4	1.89	0.28	0.47	0.43
CUPVEST2831	(AAG)_7_	154–171	9	4.27	0.30	0.77	0.74
CUPVEST3015	(TTCAA)_4_	127–158	9	3.49	0.26	0.71	0.67
CUPVEST3655	(AAG)_7_	164–181	8	2.83	0.38	0.65	0.60
CUPVEST3826	(CGG)_5_	83–136	14	3.97	0.35	0.75	0.72
CUPVEST3917	(AGAAG)_4_	129–145	6	2.51	0.23	0.60	0.52
CUPVEST3927	(TAG)_7_	147–179	20	12.31	0.61	0.92	0.92
CUPVEST3929	(TGAG)_5_	123–136	9	2.54	0.45	0.61	0.56
CUPVEST4033	(TTGT)_5_	164–179	11	4.01	0.40	0.75	0.71
CUPVEST4068	(GAT)_8_	138–167	10	3.79	0.55	0.74	0.70
CUPVEST4100	(GTTTA)_4_	140–241	11	3.73	0.50	0.73	0.67
CUPVEST4140	(CTCA)_5_	153–168	6	1.75	0.17	0.43	0.41
CUPVEST4279	(GGGGA)_4_	165–179	7	3.82	0.23	0.74	0.70
CUPVEST4680	(ATCATA)_4_	165–183	4	2.15	0.27	0.53	0.43
CUPVEST4746	(ATG)_7_	142–160	8	3.67	0.54	0.73	0.68
CUPVEST5225	(CAT)_8_	126–165	6	1.48	0.15	0.32	0.32
CUPVEST5301a	(TGGGG)_4_	161–171	5	3.38	0.43	0.70	0.66
CUPVEST534	(TTGTT)_4_	116–127	6	2.35	0.50	0.57	0.54
CUPVEST5726	(ATCAC)_5_	149–175	9	4.96	0.38	0.80	0.77
CUPVEST5746	(GAAGG)_4_	99–123	5	3.46	0.23	0.71	0.66
CUPVEST5786	(CTC)_7_	146–162	6	3.64	0.37	0.73	0.68
CUPVEST5836	(TCA)_8_	124–145	7	2.17	0.19	0.54	0.50
CUPVEST5852	(AACCCT)_4_	128–147	6	1.53	0.16	0.34	0.31
CUPVEST6009	(CTTTTT)_4_	112–135	12	4.13	0.48	0.76	0.74
CUPVEST6106	(AGA)_7_	131–146	5	3.28	0.34	0.70	0.65
CUPVEST6113	(GAA)_7_	109–124	5	2.67	0.85	0.63	0.55
CUPVEST6536	(GAAGAT)_4_	136–161	6	2.49	0.36	0.60	0.52
CUPVEST6656	(AAAT)_6_	115–123	6	3.38	0.42	0.70	0.68
CUPVEST6662	(TTG)_8_	181–187	4	2.34	0.26	0.57	0.48
CUPVEST6733	(GAA)_7_	103–179	4	2.37	0.34	0.58	0.48
CUPVEST6938	(ACG)_7_	172–179	5	2.93	0.48	0.66	0.59
CUPVEST7025	(CT)_10_	157–171	7	4.57	0.49	0.78	0.74
CUPVEST7130	(GTGAGT)_4_	126–145	5	2.82	0.59	0.65	0.60
CUPVEST7232	(GTGGA)_4_	151–162	6	3.13	0.41	0.68	0.63
CUPVEST8057	(CAA)_7_	144–177	12	4.75	0.40	0.79	0.77
CUPVEST8360	(TAG)_7_	119–134	6	4.15	0.36	0.76	0.73
CUPVEST8592	(AAGGGA)_4_	166–174	6	2.09	0.33	0.52	0.47
CUPVEST8600	(TGATT)_5_	160–178	12	2.61	0.63	0.62	0.60
CUPVEST8824	(TTTCT)_4_	146–167	10	4.78	0.41	0.79	0.76
CUPVEST8845	(GATAAG)_4_	146–171	13	2.21	0.43	0.55	0.52
CUPVEST901	(TTTCTT)_4_	134–150	7	3.35	0.27	0.70	0.65
CUPVEST9032	(CTCA)_5_	96–116	6	1.36	0.25	0.26	0.25
**SSR loci**	**Repeat motifs**	**Allele ranges (bp)**	**Na**	**Ne**	**Ho**	**He**	**PIC**
CUPVEST9042	(AGT)_7_	136–165	7	3.03	0.44	0.67	0.62
CUPVEST921	(CAGAGC)_4_	140–165	5	2.77	0.40	0.64	0.6
CUPVEST9273	(AGAAGG)_4_	126–144	7	2.17	0.23	0.54	0.49
CUPVEST9343	(TTC)_7_	124–138	7	2.85	0.21	0.65	0.58
Total			434				
Mean			7.89	3.40	0.39	0.65	0.61

In *P. vera*, a total of 150 alleles were amplified by 55 eSSR loci. The polymorphism rate was 92.7%. A total of 51 eSSR loci were polymorphic, whereas four loci were monomorphic. The average and the highest numbers of alleles were calculated as 2.73 and 6.00 (CUPVEST1313 and CUPVEST4033), respectively. The highest effective number of the allele (Ne = 4.94) was obtained from the CUPVEST4033 locus. The averages of expected heterozygosity, observed heterozygosity, and PIC values were calculated as 0.40, 0.38, and 0.34, respectively (Table 3 and Supplementary Table 12).

In *P. khinjuk*, a total of 152 alleles were produced by 52 polymorphic and 3 monomorphic eSSR loci with 94.6% polymorphism rate. An average of 2.76 alleles were obtained from 52 polymorphic loci, and the highest number of alleles (Na = 5) was in the CUPVEST3826 and CUPVEST4033 loci. The effective number of alleles ranged from 1.00 to 3.85, with an average of 2.17. The highest He (0.74) value was calculated in the CUPVEST1313 and CUPVEST3826 loci. The averages of Ho, He, and PIC values were 0.61, 0.49, and 0.42, respectively (Table 3 and Supplementary Table 13).

The lowest polymorphism (61.8%) was in *P. eurycarpa* with 34 polymorphic SSR loci. The number of alleles ranged from 1 to 4, with a total of 105 alleles. The highest values for Na, Ne, and He in *P. eurycarpa* were obtained from the CUPVEST1313 locus. The expected heterozygosity and PIC values were calculated as 0.33 and 0.27, respectively (Table 3 and Supplementary Table 14).

*Pistacia atlantica* had the highest number of alleles among *Pistacia* species in this study. A total of 191 alleles were obtained from 52 polymorphic and three monomorphic SSR loci. The CUPVEST3927 locus amplified the highest number of allele and the effective number of the allele. The averages of Na and Ne values were 3.47 and 2.12, respectively. The highest Ho value (Ho = 0.92) in *P. atlantica* was calculated in CUPVEST8600 locus, whereas the highest expected heterozygosity (He = 0.88) was calculated in the CUPVEST3927 locus. The expected heterozygosity value ranged from 0.00 to 0.88, with an average of 0.40. The average of PIC value was calculated as 0.36 (Table 3 and Supplementary Table 15).

In *P. mutica*, 117 alleles were obtained from 36 polymorphic SSR loci and 19 monomorphic SSR loci in five accessions. The mean Na and Ne values were determined as 2.13 and 1.71, respectively. The highest observed and expected heterozygosities were calculated as 0.30. The average PIC value was 0.25 in *P. mutica* (Table 3 and Supplementary Table 16).

In *P. atlantica* × *P. integerrima* (UCB1) hybrids, 39 of the 55 eSSR loci were polymorphic, with 70.9% polymorphism. A total of 109 alleles were produced by 55 eSSR loci. The highest number of the allele was obtained from the CUPVEST1146, CUPVEST4746, and CUPVEST8824 loci. The average number of allele and the effective number of allele values were 1.98 and 1.83, respectively. The highest He value was generated by CUPVEST1146, CUPVEST4746, and CUPVEST8824 loci. The average Ho and He values were calculated as 0.56 and 0.36, respectively. The average PIC value in UCB1 seedings was 0.30 (Table 3 and Supplementary Table 17).

In *P. integerrima*, the number of alleles ranged from 1 to 4. A total of 104 alleles were produced by 55 eSSR loci with 69.1% polymorphism. The average number of alleles was calculated as 1.89. The CUPVEST6536 locus amplified the highest effective number of the allele with 3.20. The highest value for He (0.69) was obtained from the CUPVEST6536 locus. The average Ho and He values were 0.63 and 0.34, respectively. The average PIC value was calculated as 0.27 in *P. integerrima* (Table 3 and Supplementary Table 18).

In *P. chinensis*, a total of 151 alleles were generated by 50 polymorphic and 5 monomorphic loci, ranging from 1 to 6 with an average 2.75 alleles per locus. The average Ne, Ho, He, and PIC were calculated as 1.82, 0.34, 0.35, and 0.31, respectively (Table 3 and Supplementary Table 19).

In *P. terebinthus*, 180 alleles were obtained from 46 polymorphic and 9 monomorphic loci. The average number of the allele was 3.27. The highest number of the alleles was obtained from the CUPVEST3927 and CUPVEST6009 loci. The average effective number of allele and the highest effective number of alleles were 2.02 and 6.54, respectively. The CUPVEST3927 SSR locus produced the highest expected heterozygosity value. The average Ho, He, and PIC values were calculated as 0.34, 0.38, and 0.33, respectively (Table 3 and Supplementary Table 20).

In *P. palaestina*, 197 alleles were generated from 34 polymorphic and 21 monomorphic loci. The average number of the allele was detected as 2.07. The average effective number of allele was 1.63. The average Ho, He, and PIC values were calculated as 0.28, 0.28, and 0.31, respectively (Table 3 and Supplementary Table 21).

In *P. lentiscus*, 38 of 55 eSSR loci were polymorphic with 69.1%. A total of 131 alleles were amplified with an average of 2.38 alleles per locus. The average effective number of the allele was detected as 1.65. The highest number of allele (Na = 7), the effective number of the allele (Ne = 3.90), observed heterozygosity (Ho = 0.92), and expected heterozygosity (He = 0.74) values were obtained from the CUPVEST3826, CUPVEST5726, CUPVEST1146, and CUPVEST5726 loci, respectively. The average values for Ho and He were 0.24 and 0.29, respectively. The average PIC value was calculated as 0.27 (Table 3 and Supplementary Table 22).

Genetic diversity values of unknown accessions were calculated as well. A total of 53 eSSR loci were polymorphic with 96.4%. The average numbers of Na, Ne, Ho, He, and PIC values were 2.96, 2.10, 0.45, 0.46, and 0.41, respectively (Table 3 and Supplementary Table 23).

### Clustering and Structure Analysis

**FIGURE 6:**
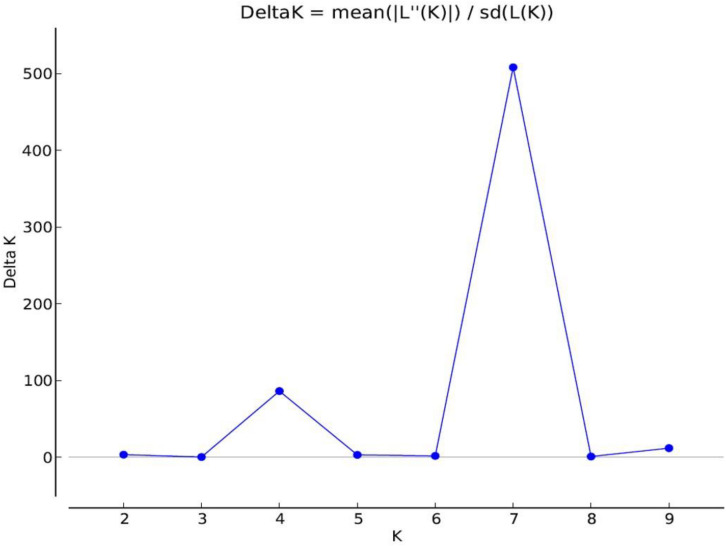
The graph demonstrates number of possible population (*K* = 7) and subpopulation (*K* = 4). *X* axis refers to number of *K*, and *Y* axis refers to Delta *K*.

**FIGURE 7:**
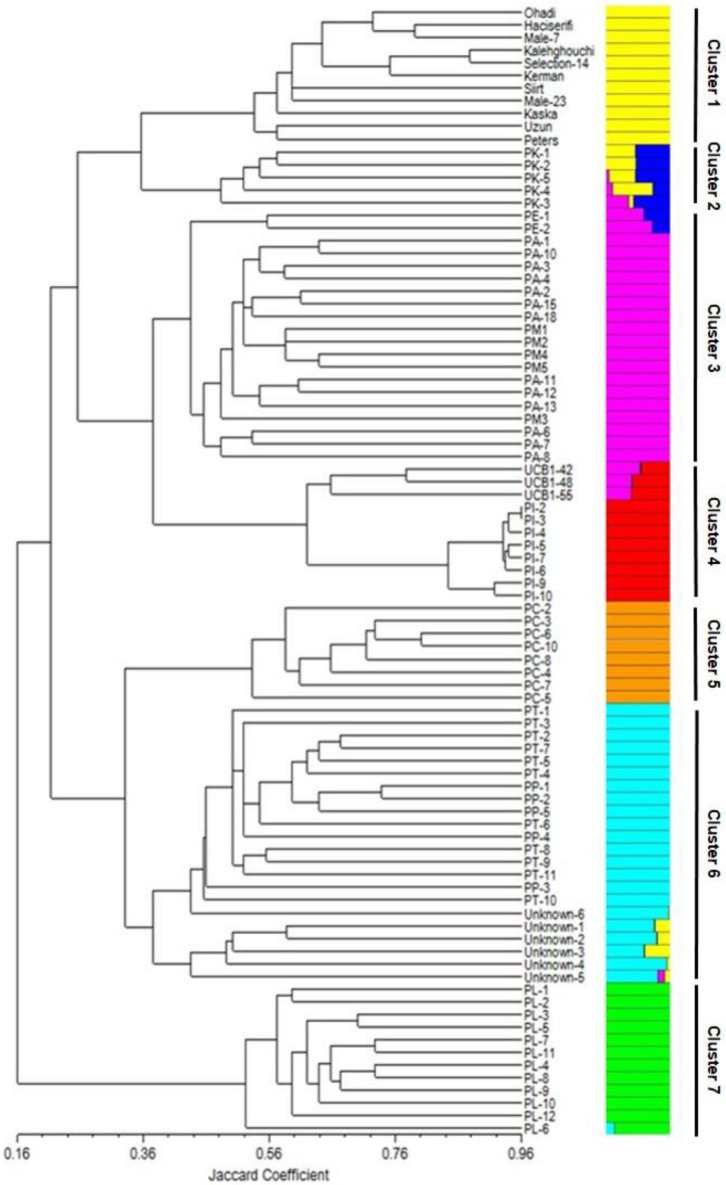
UPGMA dendrogram of 11 *P. vera* and 78 wild *Pistacia* genotypes belongs to *P. khinjuk*, *P. eurycarpa*, *P. atlantica*, *P. mutica*, UCB1, *P. integerrima*, *P. chinensis*, *P. terebinthus*, *P. palaestina*, and *P. lentiscus*. The figure refers to the number of possible populations at *K* = 7 (Delta *K* = 7).

**FIGURE 8:**
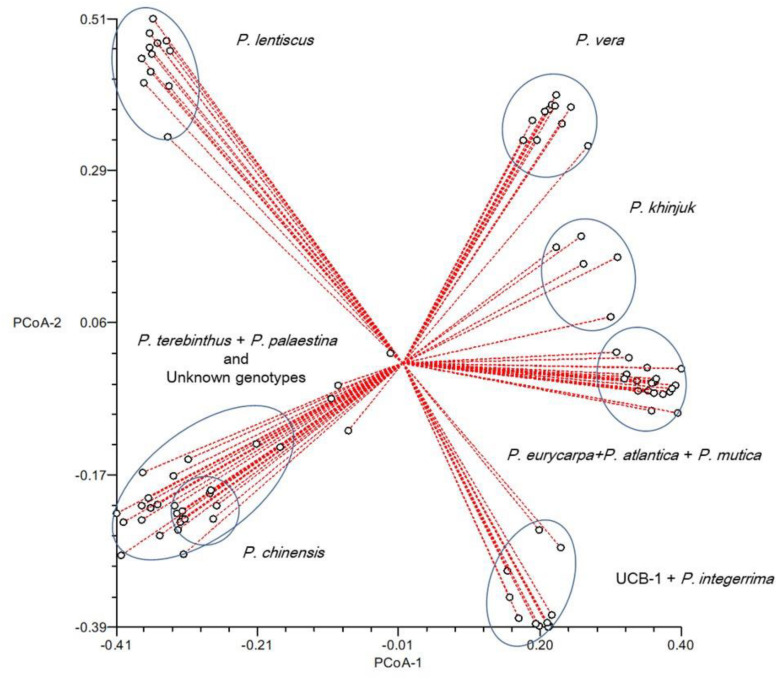
Principal coordinate analysis of the 89 *Pistacia* individuals.

The maximum Delta *K* value was at *K* = 7 (Figure 6). *Pistacia* accessions were grouped in seven main clusters: Cluster 1 included *P. vera* cultivars, whereas *P. khinjuk* was the closest species to *P. vera*. *P mutica* and *P. atlantica* were in Cluster 3 together with *P. eurycarpa*, which was clearly separated from *P. mutica* and *P. atlantica*. Cluster 4 included *P. integerrima* and UCB1 (*P. atlantica* × *P. integerrima*) accessions. Three UCB1 accessions were clearly separated from *P. integerrima*. *P. chinensis* accessions were in Cluster 5. *P. terebinthus* and *P. palaestina* accessions were grouped in the same cluster (Cluster 6) together with unknown *Pistacia* accessions. Cluster 7 consisted *P. lentiscus* accessions that were separated from rest of the accessions in the UPGMA analysis (Figure 7). A PCoA supported the structure and UPGMA analysis (Figure 8).

## Discussion

### Identification of eSSRs

The density of eSSRs distribution was computed as one SSR per 17.75 kb in the present study and was higher than other species such as *Argyranthemum brousonetii* (2.3%, 27.00 kb) and *Zingiber officinale* (2.7%, 25.20 kb) (White et al., 2016; Awasthi et al., 2017), while it was lower than in some species such as *Arachis hypogaea* (17.7%, 3.30 kb), *Curcuma longa* (20.5%, 4.80 kb), and *Curcuma alismatifolia* (12.5%, 6.60 kb), respectively (Annadurai et al., 2013; Wang et al., 2018; Taheri et al., 2019). eSSR frequencies and their density in genomes may differ from species to species, because each species has different genetic construction. On the other hand, using different bioinformatics tools and criteria for detection of SSRs may also be a reason for the differences (Liu et al., 2018; Taheri et al., 2019).

eSSR repeat motifs identified from dinucleotide to hexanucleotide and trinucleotide repeats (29.54%), dinucleotide repeats (24.06%), and hexanucleotide repeats (20.67%) were the most abundant repeat motifs, respectively. These results were similar to those found in previous studies in abundance of trinucleotide motifs within the transcriptome sequences (Awasthi et al., 2017; Park et al., 2019; Taheri et al., 2019). A previous study in *P. vera* by Jazi et al. (2017) also demonstrated that dinucleotides (44.7%) and trinucleotides (40.6%) were the most abundant types of repeats. The most frequent types in genic SSRs are trinucleotide repeat type, whereas the common types of SSRs in unigene sequences are dinucleotide and trinucleotide types (Varshney et al., 2002).

### Transcriptome Assembly and Functional Annotation

*De novo* sequencing and assembly without aligning with the reference genome have been widely used to obtain first sequences for non-model organisms (Russell et al., 2014; Wei et al., 2014; Chen et al., 2017). The transcriptome sequences of *P. vera* were the first provided by Jazi et al. (2017) for discovery of markers about salinity-related genes. In the present study, different tissues of a female and a male *P. vera* cultivars were sequenced, and N50 of unigenes was computed as 1,333 bp, similar with the study performed by Jazi et al. (2017).

The GO database is one of the largest information sources about detection of the genes functions ranging from model organisms to minor organisms (Gene Ontology Consortium, 2004). For GO analysis, a total of 40,405 sequences were associated with 325,220 GO terms, which classified different 56 subcategories in three major categories in this study. Jazi et al. (2017) demonstrated that 68,539 sequences were annotated with 302,375 GO terms. The results indicated that assembled unigenes have different molecular functions involved in different metabolic pathways. The assembled sequences were aligned to COG database for prediction of the possible functions. KO provides recognition of the biological pathway of transcripts using KEGG database (Blanca et al., 2011; Torre et al., 2014). Therefore, KEGG pathway analysis explains the information about biological systems of organisms and the relationships between transcripts and their molecular, cellular, and organism levels (Kanehisa et al., 2008). A total of 30,746 unigenes were associated with 279 KEGG pathways. The results illustrated that the KEGG pathway classification of the *P. vera* will facilitate to understand related complex traits at transcriptome level in pistachio and in closely related species.

### eSSR Polymorphism in *Pistacia*

SSR markers have been widely preferred for genetic diversity studies, construction of consensus genetic linkage maps, QTL mapping, and MAS in breeding programs (Li et al., 2012; Dong et al., 2018). SSR markers from transcriptome sequences are especially valuable because of their preserved gene regions (Taheri et al., 2019). NGS provides more opportunities than detecting classic SSRs and generates enormous data for development of SSRs in many plant species including *P. vera* (Jazi et al., 2017). The detection of potential SSR markers in pistachio is very easy owing to its high heterozygosity rate (Motalebipour et al., 2016; Jazi et al., 2017). Although Jazi et al. (2017) detected 11,337 potential SSRs in *P. vera*, a total of 14,308 genic SSR loci were determined in this study.

In *P. vera*, several reports were published regarding the development of novel genomic SSR markers in pistachio. First, novel 14 SSR markers were developed by Ahmad et al. (2003) in *P. vera*. Kolahi-Zonoozi et al. (2014) designed 42 primers from Akbari pistachio cultivar and selected 12 polymorphic SSR loci in 45 economically important pistachio cultivars. Then, Zaloğlu et al. (2015) constructed genomic libraries enriched with dinucleotides and trinucleotides repeats. They developed 47 polymorphic SSR loci from *P. vera* cv. Siirt. Topçu et al. (2016) sequenced 192 clones from enriched (GA)_n_ and (AAG)_n_ motifs. In total, 110 of 135 primers were produced in PCRs, and 64 of them were found polymorphic with 264 alleles in 12 pistachio cultivars. Currently, there are a few studies about the development of genomic SSR markers in *Pistacia* using NGS technology (Motalebipour et al., 2016; Khodaeiaminjan et al., 2018). Motalebipour et al. (2016) developed SSR markers from *P. vera*, and their transferability was tested in five wild *Pistacia* species. Khodaeiaminjan et al. (2018) developed 625 polymorphic *in silico* SSR markers. We developed here genic SSR markers using NGS and generated 33,341 SSR loci.

Motalebipour et al. (2016) developed 204 SSR markers and tested them in five wild *Pistacia* species. The means of Ho, He, and PIC values in 24 *P. vera* accessions were calculated as 0.46, 0.55, and 0.50, respectively (Motalebipour et al., 2016). Khodaeiaminjan et al. (2018) used 613 *in silico* SSR loci with an average number of allele 4.2 in 18 *P. vera* genotypes. Average values for observed (Ho) and expected (He) heterozygosities and PIC were 0.53, 0.56, and 0.51, respectively, in 18 *P. vera* cultivars. In this study, we used 55 eSSR loci with an average number of 2.73 allele in only *P. vera*.

There are a limited number of studies about development of SSR markers in wild *Pistacia* species. Albaladejo et al. (2008) developed novel SSR markers from *P. lentiscus* using enriched library method and produced 59 alleles from eight SSR markers, ranging from 3 to 13 per locus, although a total of 131 alleles were generated by 55 eSSR loci in *P. lentiscus* genotypes in this study. Arabnezhad et al. (2011) designed 27 primer pairs enriched for dinucleotide (AG)_n_ and trinucleotide (ATG)_n_ motifs from *P. khinjuk* sequences. A total of 114 alleles were generated with an average number of allele Na = 2.8 per locus in all *Pistacia* accessions (Arabnezhad et al., 2011). In this study, 52 of 55 eSSRs were found polymorphic, and a total of 152 alleles were generated with an average number of allele Na = 2.8 in five *P. khinjuk* accessions.

### Phylogeny of *Pistacia* Species

In this study, structure analysis separated *Pistacia* accessions in seven main clusters. *P. khinjuk* was the closest species to cultivated *P. vera* as in previous studies (Zohary, 1952; Kafkas and Perl-Treves, 2001, 2002; Golan-Goldhirsh et al., 2004; Al-Saghir and Porter, 2006; Kafkas, 2006a; Motalebipour et al., 2016). *P. lentiscus* was the most distant species to *P. vera* in accordance with previous studies (Zohary, 1952; Kafkas and Perl-Treves, 2001, 2002; Golan-Goldhirsh et al., 2004; Kafkas, 2006a,b; Motalebipour et al., 2016).

Zohary (1952) defined that *P. eurycarpa* is a subspecies of *P. atlantica* (var. *kurdica*), whereas it was classified as a different species by Yaltırık (1967). In this study, we used two old *P. eurycarpa* accessions sampled from Göbek village of Gaziantep province to emerge relationship with other *Pistacia* species. The results clearly demonstrated that *P. eurycarpa* was closer to *P. atlantica* than *P. khinjuk*. Kafkas and Perl-Treves (2001) found that *P. atlantica* and *P. eurycarpa* were closely related species at molecular level. Therefore, the results in this study supported statements at morphological level by Yaltırık (1967) and at molecular level by Kafkas and Perl-Treves (2001).

Al Yafi (1978) detected different subspecies of *P. atlantica* using some leaf characters. He reported that *P. mutica* had been a subspecies in *P. atlantica*. Kafkas (2006a) described that *P. atlantica* and *P. mutica* were not clearly diverged. Therefore, *P. mutica* was grouped within *P. atlantica*. In this study, pairs of species were not prominently separated from each other. The previous findings supported that *P. atlantica* and *P. mutica* species were one of the closest pairs of species (Al-Saghir and Porter, 2006; Kafkas, 2006a).

*P. integerrima* and its hybrids have been used as rootstock for *P. vera* in California. Zohary (1952) defined *P. integerrima* as a subspecies of *P. chinensis*, whereas Parfitt and Badenes (1997) classified it as a distinct species. In this study, *P. integerrima* and *P. chinensis* were clearly separated from each other. Similar results were also obtained by Kafkas and Perl-Treves (2001), Kafkas (2006a), and Motalebipour et al. (2016).

There is still discussion about whether *P. terebinthus* and *P. palaestina* are same or different species. The first classification was performed by Engler (1883), who considered *P. palaestina* as a variety of *P. terebinthus*. However, Zohary (1952) described that *P. palaestina* was a different species from *P. terebinthus*. On the other hand, Yaltırık (1967) reported that *P. palaestina* was a subspecies of *P. terebinthus.* Kafkas and Perl-Treves (2001) and Kafkas (2006a) supported Yaltirik and Engler’s classification studies. In this study, these species were not prominently diverged from each other. Therefore, our hypothesis is that they are same species, and *P. palaestina* can be a subspecies of *P. terebinthus*.

In this study, six unknown *Pistacia* genotypes were grouped with *P. palaestina* and *P. terebinthus* according to UPGMA analysis. Structure analysis demonstrated that they have close relationships with cultivated *P. vera*. This situation can be described that these genotypes may be hybrids between *P. vera* and *P. palaestina* or *P. terebinthus*.

## Conclusion

Transcriptome sequencing provided opportunities for mining easy and cost-effective SSR markers using NGS platform. A total of 98,831 unigenes in this study can be useful for genome annotation in *P. vera* and in related species in the future. The SSR distribution frequency in pistachio transcriptome was one SSR per 17.75 kb. A total of 14,308 eSSRs were defined using transcriptome data of pistachio, and they can be used in studies such as germplasm characterization, population and evolutionary studies, marker-assisted breeding, and association and QTL mapping in *Pistacia* species. This was the first study characterizing 10 *Pistacia* species by genic SSRs and provided important findings on the taxonomy of *Pistacia* species.

## Data Availability Statement

The sequence data have been deposited in the National Center for Biotechnology Information (NCBI) under BioProject accession number PRJNA648340.

## Author Contributions

HK, EI, and SK prepared plant materials. HK, AP, HT, and SK performed the bioinformatic and SSR analysis. HK, AP, and SK wrote the manuscript. All the authors contributed to the article and approved the submitted version.

## Conflict of Interest

The authors declare that the research was conducted in the absence of any commercial or financial relationships that could be construed as a potential conflict of interest.
